# Is polyendocrine metabolic ovarian syndrome a legacy of excessive gestational weight gain? A review of developmental and transgenerational programming

**DOI:** 10.3389/fendo.2026.1978407

**Published:** 2026-10-30

**Authors:** Zhiwen Ge, Jie Wang, Chen Zhang

**Affiliations:** 1 Department of the First School of Clinical Medicine, Zhejiang Chinese Medical University, Hangzhou, Zhejiang, China; 2 Department of Gynecology and Obstetrics, The First Affiliated Hospital of Zhejiang Chinese Medical University, Hangzhou, Zhejiang, China

**Keywords:** endocrine programming, excessive gestational weight gain, fetal programming, hypothalamic-pituitary-ovarian axis, placental dysfunction, polycystic ovary syndrome, polyendocrine metabolic ovarian syndrome

## Abstract

Polyendocrine Metabolic Ovarian Syndrome (PMOS), formerly known as Polycystic Ovary Syndrome (PCOS), is increasingly recognized as a systemic endocrine-metabolic disorder characterized by hyperandrogenism, ovulatory dysfunction, insulin resistance, adipose tissue dysfunction, and hypothalamic-pituitary-ovarian (HPO) axis alterations. Evidence suggests susceptibility may originate before reproductive maturity, potentially during fetal development. This review examines whether excessive gestational weight gain (EGWG) elevates offspring PMOS risk through developmental (intergenerational and, potentially, transgenerational) endocrine programming. EGWG, defined as weight gain above the recommended range for prepregnancy body mass index, may reflect heterogeneous changes in maternal and fetal tissues rather than excess maternal fat accumulation alone. In some pregnancies, EGWG co-occurs with insulin resistance, altered lipid metabolism, inflammation, or adipokine dysregulation. These changes could modify placental nutrient transfer and endocrine signaling, thereby influencing offspring metabolic and reproductive development. However, the pathways linking EGWG specifically to PMOS remain uncertain. Human studies associate maternal metabolic conditions and PMOS with selected offspring outcomes, but provide limited evidence that EGWG independently predicts PMOS in daughters. Experimental models show that prenatal androgen exposure and maternal dietary perturbations can produce PCOS-like reproductive and metabolic traits, supporting the biological plausibility of developmental programming. This review evaluates whether EGWG may act as a marker or modifier of intrauterine conditions relevant to later PMOS susceptibility, while considering prepregnancy adiposity, maternal PMOS, genetic predisposition, and postnatal exposures as alternative or interacting explanations. Prospective mother–daughter cohorts with repeated pregnancy measures, placental profiling, and follow-up through puberty and adulthood are needed to test this hypothesis.

## Introduction

Polyendocrine Metabolic Ovarian Syndrome (PMOS) has been known traditionally as Polycystic Ovary Syndrome (PCOS). It is among the most common and biologically varied endocrine diseases that impact women of childbearing years (1). The syndrome was previously described based upon ovarian morphology alone. Therefore, the syndrome cannot be defined simply as the result of “cysts.” PMOS defines a disorder involving endocrine, metabolic, reproductive, and systemic regulation (2). The new nomenclature recognizes that this is a syndrome involving multiple hormonal axes, with metabolic dysfunction (specifically insulin resistance, adipose tissue dysregulation, and long-term cardiometabolic risk) as a central component (3). This change has clinical relevance because it reframes what were perceived as primarily ovarian issues as a multisystem endocrine-metabolic syndrome with potential developmental, reproductive, and intergenerational consequences (4). Recent nomenclature initiatives have proposed Polyendocrine Metabolic Ovarian Syndrome as a replacement term for PCOS; however, most indexed literature and current clinical criteria still use PCOS terminology (5). Throughout this review, we interpret evidence reported under the older PCOS terminology as informing the PMOS framework while recognising that PCOS-derived findings do not automatically validate every component of the newer PMOS construct.

PMOS has a substantial global impact. The World Health Organization has estimated that about 10–13 percent of women of childbearing years will develop PCOS; in addition, up to 70% of those who do develop PCOS can go for many years without being diagnosed (6). Most commonly PCOS presents with symptoms such as irregular menstrual cycles, anovulation (lack of ovulation), difficulty conceiving, excessive hair growth (hirsutism), acne, obesity, and increased blood glucose levels due to insulin resistance, as well as emotional/psychological distress (7). In most cases, PMOS begins to manifest itself at puberty. Nevertheless, the underlying mechanisms may begin during fetal development, neonatal life, early childhood (when fat stores expand), or pubertal endocrine maturation (8). A developmental approach matters because it shifts the focus from treating the manifestations of reproductive and metabolic susceptibility toward understanding how that susceptibility arose (9).

The growing interest in EGWG within developmental endocrinology reflects the fact that pregnancy is a period when maternal metabolic health directly shapes the fetal environment (10). EGWG may be accompanied by a chronic, low-grade inflammatory and endocrine state characterised by insulin resistance, hyperinsulinemia, altered lipid profiles, dysregulated adipokine secretion, oxidative stress, mitochondrial strain, and placental metabolic adaptations (11). These changes can modify nutrient delivery, steroid exposure, and inflammatory signalling to the fetus during critical windows of organ development (12). Female offspring are exposed to these windows at different developmental stages, including ovarian germ-cell formation, primordial follicle assembly, granulosa-theca cell communication, maturation of the hypothalamic-pituitary-ovarian (HPO) axis, establishment of adipose tissue patterning, pancreatic beta-cell adaptation, and hepatic insulin sensitivity (13). Disruption of these systems can predispose females to the reproductive and metabolic features of PMOS (14).

The biological link between EGWG and offspring PMOS risk is strengthened by the overlap between obesity-associated metabolic changes in pregnancy and the key mechanisms of PMOS (9). Insulin resistance is a central feature of many forms of PMOS: it promotes ovarian androgen production by stimulating theca-cell steroidogenesis and lowering sex hormone-binding globulin (SHBG), which increases free androgen availability (15). Exposure of the developing fetus to this excess of insulin, glucose, fatty acids, inflammatory mediators, and altered steroid signals may therefore reshape ovarian steroidogenic pathways, hypothalamic gonadotropin-releasing hormone (GnRH) pulsatility, adipose tissue function, and insulin sensitivity (16). These alterations establish a developmental context in which PMOS symptoms emerge when the child reaches puberty. Puberty is therefore unlikely to be the initiating event in PMOS pathogenesis; rather, it is the stage at which earlier endocrinologically programmed changes become clinically apparent (17).

The placenta is central to this pathway. As an endocrine, immune, vascular, and nutrient-sensing organ, it regulates fetal exposure to metabolic signals from the mother (18). In pregnancies complicated by obesity, placental function can be altered through inflammation, oxidative stress, mitochondrial dysfunction, changes in lipid transport, altered glucose handling, and disrupted steroid metabolism (19). The programming model therefore must consider not only maternal ovaries and adipose tissue but also the integrated maternal-placental-fetal endocrine unit (20).

Evidence of relationships among EGWG, maternal PMOS (formerly PCOS), offspring adiposity, offspring cardiometabolic disturbances, and offspring reproductive disorders are demonstrated using data from human cohort studies (21). However, human-based research is difficult to interpret because EGWG frequently coexists with a genetic predisposition to obesity, maternal gestational diabetes, maternal hyperandrogenemia, shared environmental exposures during development (e.g., diet), socioeconomic status, and a postnatal obesogenic environment (22). Animal model experiments provide additional mechanistic insights into how prenatal androgen exposure, maternal high-fat diet, and obesogenic environments contribute to PMOS like phenotypes in offspring. These models have also been shown to alter neuroendocrine function, decrease insulin sensitivity, create adipose dysfunction, increase oxidative stress, and alter gene expression (23).

The distinction between intergenerational and transgenerational effects is central to interpreting the evidence. When a pregnant woman (F0) experiences excessive gestational weight gain (EGWG), her fetus (F1) is exposed to the associated intrauterine environment; the fetus’s developing germ cells, which may later give rise to F2, may also be affected (24). For transgenerational inheritance, phenotypic changes must persist for at least one generation beyond the generation directly exposed to EGWG (25). This typically involves germline-associated epigenetic mechanisms, including DNA methylation, histone modifications, non-coding RNA, mitochondrial dysfunction, and chromatin remodeling (24, 25). Consistent with the intergenerational scope of this Research Topic, we focus on the directly exposed F1 (and, where relevant, F2) generations; in humans, the available evidence largely documents intergenerational (F1) exposure, whereas true transgenerational inheritance has been demonstrated mainly in appropriately designed animal and germline studies.

This review asks whether part of the cause of PMOS can be attributed to a legacy of EGWG through developmental endocrine programming, while treating EGWG as a potentially modifiable developmental amplifier rather than a sole cause (26). This review integrates evidence on placental biology, ovarian development, neuroendocrine regulation, metabolic inheritance, and epigenetic transmission to evaluate how prenatal endocrine disruption may influence lifelong PMOS susceptibility and identify opportunities for prevention (27).

## Search methodology

This review is a mechanism-driven narrative synthesis with systematic search components that evaluates if EGWG increases the likelihood of an offspring’s polyendocrine metabolic ovarian syndrome risk by altering developmental, endocrine, metabolic, placental and epigenetic programming. The topic has the potential for integration of human epidemiological studies, experimental biological research, reproductive endocrinology studies, placental sciences, and trans-generational mechanisms. Thus, a meta-analysis was not deemed appropriate because of limited studies searched, anticipated variability in definition of exposure, diagnostic criteria used, animal or cell culture models used, outcomes measured, and generational endpoint studied.

An organized search across multiple databases (PubMed/MEDLINE; Scopus; Web of Science; Embase; Google Scholar; Cochrane Library; ClinicalTrials.gov; and guideline repository) was performed. The database searches used an updated/legacy term combination in order to identify both recent literature on polyendocrine metabolic ovarian syndrome and a larger amount of literature on polycystic ovary syndrome. In addition to “polyendocrine metabolic ovarian syndrome”, “polycystic ovary syndrome”, “PCOS”, “PMOS”, “excessive gestational weight gain”, “gestational weight gain”, “pre-pregnancy BMI”, “maternal high fat diet”, “offspring”, “daughter”, “fetal programming”, “developmental programming”, “intergenerational”, “transgenerational”, “prenatal androgen”, “hyperandrogenism”, “insulin resistance”, “placenta”, “folliculogenesis”, “ovarian development”, “adipokines”, “epigenetic inheritance”, “DNA methylation”, “non-coding RNA”, “germline transmission” were also searched as search terms. Boolean operators were used in conjunction with PCOS/PMOS terms along with maternal metabolic exposure terms and developmental outcome terms. The search was conducted up to July 2026 and was restricted to English-language publications. Screening of titles, abstracts, and full texts was performed independently by two reviewers, with disagreements resolved by consensus or by a third reviewer. Because this is a hybrid narrative review with systematic search components rather than a formal systematic review, formal risk-of-bias and quality appraisal tools were not applied; however, methodological quality was considered when assessing eligibility. The review was reported in accordance with the PRISMA 2020 statement where applicable.

The screening pathway is illustrated in **Figure 1**. Records were located through a search of literature databases (n=1246) and registers (n=78), with inclusion of Cochrane reviews and clinical trials. Duplicate records (n=412) were removed from this number prior to an assessment of the remaining 912 titles and abstracts. Excluded were 720 titles and abstracts based on a determination that their content was not relevant to EGWG, PMOS/PCOS, developmental programming, or offspring endocrine-metabolic outcome. In order to assess eligibility for inclusion in the synthesis, 192 articles were requested as full text. Seven articles were unable to be obtained, resulting in the assessment of 185 articles for eligibility. One hundred articles were excluded because they were not mechanistic studies, had limited sample sizes or poor methodological quality, or represented duplicate data or review-only evidence. Therefore, 85 studies were synthesized.

**Figure 1:**
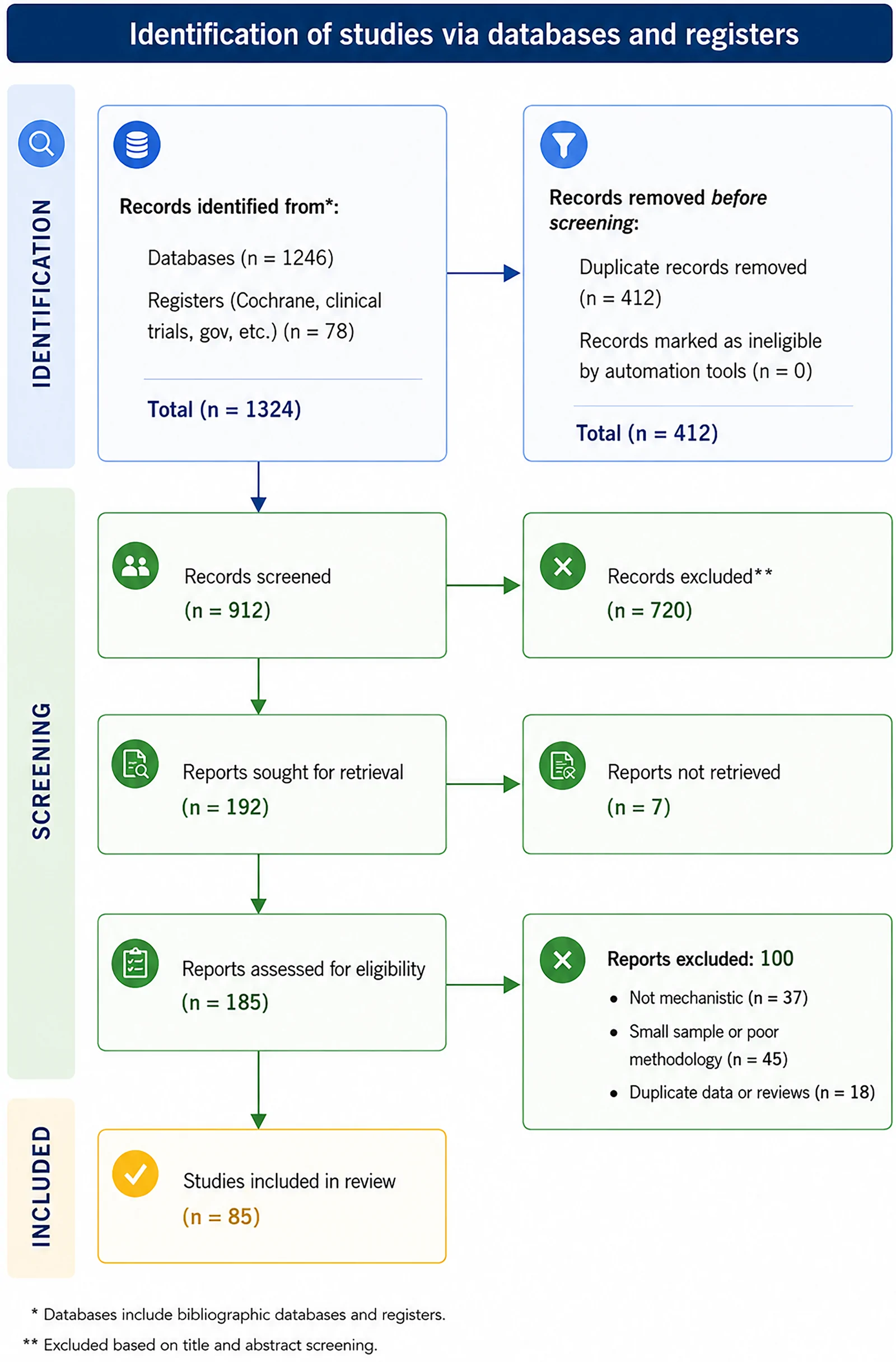
PRISMA flow diagram of study identification, screening, eligibility assessment, and final inclusion of 85 studies.

Data extraction was based on maternal exposure type, offspring sex, developmental time frame, study design, model system, ovarian outcomes, metabolic outcomes, placentas, androgen mechanisms, epigenetic markers, and the strength of transgenerational inference. The studies were then synthesized into thematic areas to show mechanistic convergence in addition to statistical aggregation.

## Excessive gestational weight gain as a gestational endocrine-inflammatory state

EGWG is more than just excessive body mass index (BMI) in pregnancy. It may be an active biological inflammatory and endocrine condition affecting the mother-placenta-fetus triad at critical stages of tissue growth and maturation (28). Insulin resistance, elevated insulin levels, lipid disturbances, alterations in adipokines, chronic subclinical inflammation, oxidative stress, mitochondrial stress, and changes in the placenta’s endocrine function are all examples of these effects (29).

### Maternal insulin resistance, lipotoxicity, and fetal nutrient oversupply

Normal pregnancy is characterized by increasing insulin resistance throughout gestation, peaking in the second and third trimesters so that more nutrients are available for fetal growth (19). However, in obese mothers, this normal physiologic adaptation can be exaggerated, thereby increasing the maternal concentration of insulin, glucose, triglycerides, and free fatty acids. Consequently, the intrauterine environment may expose the fetus to an abundance of glucose and lipids that can disrupt the developmental pathways governing pancreatic beta-cell function, hepatic lipid metabolism, muscle insulin sensitivity, adipose tissue growth, and ovarian metabolic signalling (30).

Lipotoxicity is particularly important because free fatty acids activate inflammation and oxidative stress, impair mitochondrial function, influence cell differentiation, and alter the cellular environment (31). Maternal diets that are high in fat have been associated in both animals and humans with excessive fetal fat deposition, increased levels of pro-inflammatory cytokines, and increased levels of fetal-free fatty acid exposure. These alterations could create a prenatally programmed or predisposed condition of decreased metabolic flexibility (making the fetus/infant more susceptible to insulin resistance) upon exposure to post-natal obesity related conditions (32). Therefore, within a PMOS framework, the metabolic vulnerability caused by prenatal programming would be mechanistically located “upstream” of ovarian hyperandrogenism (due to direct and indirect effects on androgen production and binding of steroids), as insulin can stimulate theca-cell production of steroids, while reducing SHBG (sex-hormone-binding globulin) thereby increasing free androgen bioavailability.

Adaptations of the fetal liver, pancreas, adipose tissue, and ovaries to nutrient excess may lower the thresholds for insulin resistance, obesity, and adult hyperandrogenism (33).

### Adipose inflammation, adipokine imbalance, and oxidative stress

For women with obesity, adipose tissue is an immune-endocrine organ that expands and releases inflammatory factors, alters the macrophage’s polarization and influences the balance of adipokines (34). Fetal exposure to increased levels of leptin, decreased levels of adiponectin and elevated levels of pro-inflammatory cytokines (TNF-α, IL-6) may affect placental function, the development of the fetal hypothalamus, fat cell production (adipogenesis), and glucose metabolism. Because these signals regulate appetite, gonadal steroidogenesis, insulin signaling, and inflammatory tone, a fetus exposed to them may acquire altered energy-balance set points and altered patterns of metabolic-reproductive communication (35).

EGWG has been linked to several mechanisms through which endocrine programming may induce oxidative stress in offspring. These include increased generation of reactive oxygen species by excess nutrient intake and inflammation; and compromised cellular energy production due to mitochondrial stress (36). Mitochondrial dysfunction in reproductive tissue may lead to decreased oocyte quality and impaired function of both granulosa cells and steroidogenesis, along with alterations in ovarian reserve dynamics. The same mechanism may cause an increase in insulin resistance and fat deposition in metabolic tissue (37). Studies of placental development have shown that EGWG leads to chronic inflammation, oxidative stress and changes in the function of the placenta, suggesting that the placenta mediates maternal metabolism rather than merely being a conduit for it.

This inflammatory oxidative environment also interacts with androgens. In PMOS hyperandrogenism and insulin resistance amplify inflammatory signaling and inflammation worsens insulin resistance and ovarian dysfunction (38). If EGWG exposes the fetus to inflammatory and oxidative signals during ovarian and neuroendocrine development offspring susceptibility may be shaped through a multi-hit process involving metabolic stress and imbalance of steroids and remodeling at the tissue level of hormones (4).

### Placental endocrine adaptation and fetal exposure windows

The placenta acts as the primary mediator for the effects of EGWG at a biological level (29). It interprets maternal metabolic signals including glucose, lipids, insulin, cytokines, oxygen levels and hormones to regulate fetal exposure via nutrient transfer across the placenta (via transport proteins), regulation of blood flow to the placenta (vascular regulation), production of immune cells or suppression of them (immune system regulation), mitochondrial energy metabolism and hormone regulation (39).

Placental adaptations are not necessarily harmful; the placenta can sometimes support fetal growth by modulating nutrient transfer and endocrine signaling. However, when maternal metabolic stress persists, the placental compensations that were originally protective may evolve into maladaptive responses (19). For example, increased lipid delivery could result in the fetus being exposed to excessive amounts of free fatty acids; inflammatory response may also modify the placenta’s vascular structure and hormone secretion. Mitochondrial failure may lower placental energy efficiency, and changes in the metabolism of steroids could increase the fetus’ exposure to active forms of androgen and/or glucocorticoid (40).

Exposure in early gestation may lead to alterations in organ formation, placental structure, and germ cells. Exposure in mid-gestation may influence the development of follicles, hypothalamic regions, and the organization of metabolically active tissues (41). Exposure in late gestation may affect fat deposition, beta-cell function (influencing insulin sensitivity), liver metabolism of lipids, and neonatal hormonal adaptation (42).

Recent single-cell and transcriptional studies of the placenta provide further evidence that obesity alters maternal-fetal interface biology through nutrient transport and developmental signaling (18).

### Excessive gestational weight gain, SHBG suppression, and androgen bioavailability

EGWG interacts with androgen physiology as well. The hyperinsulinaemia associated with EGWG reduces hepatic sex hormone-binding globulin (SHBG) levels which in turn increases the levels of the bioavailable fraction of free androgens (43) (**Table 1**). Therefore, pregnancies complicated by both EGWG and maternal PMOS may expose the fetus to a doubly metabolically-androgenically challenged fetal environment (27).

**Table 1:** Mechanistic pathways linking EGWG to fetal endocrine, metabolic, placental, and ovarian targets.

Maternal obesity-related exposure	Primary mediator	Fetal or offspring target	PMOS-relevant programmed effect
Maternal insulin resistance (43)	Hyperinsulinemia, altered glucose flux	Fetal pancreas, liver, skeletal muscle, ovary	Lower threshold for insulin resistance and insulin-mediated androgen amplification
Dyslipidemia and lipotoxicity (33)	Free fatty acids, triglycerides, lipid-derived inflammatory signals	Placenta, fetal liver, adipose tissue, ovarian cells	Metabolic inflexibility, adiposity, mitochondrial stress, altered steroidogenic environment
Adipose inflammation (31)	TNF-α, IL-6, CRP, macrophage activation	Placenta, hypothalamus, adipose tissue, ovary	Chronic inflammatory tone, disrupted insulin signaling, altered reproductive-metabolic communication
Adipokine imbalance (16)	Increased leptin, reduced adiponectin, altered appetite signaling	Hypothalamus, adipose tissue, ovarian microenvironment	Leptin resistance, adipose expansion, impaired metabolic regulation, possible ovarian dysfunction
Oxidative and mitochondrial stress (46)	Reactive oxygen species, impaired mitochondrial efficiency	Placenta, oocytes, granulosa cells, metabolic tissues	Reduced cellular metabolic resilience, impaired follicular support, insulin resistance susceptibility
Placental maladaptation (47)	Altered nutrient transport, vascular function, steroid metabolism, immune signaling	Maternal-fetal interface and fetal endocrine organs	Modified fetal exposure to nutrients, inflammatory mediators, and steroid hormones
Reduced SHBG and altered androgen bioavailability (48)	Hyperinsulinemia, increased free androgen fraction	Fetal ovary, HPO axis, adipose tissue	Androgen-sensitive programming of folliculogenesis, LH regulation, and later hyperandrogenism
Combined maternal PMOS and obesity (27)	Metabolic-androgenic co-exposure	Ovary, hypothalamus, liver, adipose tissue, germline	Higher probability of offspring reproductive-metabolic phenotype expression

Animal models are an important source of evidence for how androgenic and obesogenic prenatal exposures may also interact. Female offspring exposed to high levels of androgens prenatally have been found to exhibit reproductive dysfunction, abnormal hormone (testosterone/LH) patterns, irregular menstrual cycles, and PCOS-like ovarian morphology; however, maternal diet-induced obesity may also affect fetal metabolic organs (23). Studies using animal models of maternal diet-induced obesity combined with high levels of androgen exposure have indicated alterations in both maternal/fetal liver function as well as placental expression of the androgen receptor, and lipid-related pathway changes indicating that obesity and androgen exposure may effect reproductive and/or metabolic programming through similar mechanisms (44).

Maternal hyperinsulinemia may program fetal pancreatic beta-cell development and insulin secretion (26). EGWG may also lower fetal SHBG, increasing circulating free testosterone, and impair placental steroid handling. Together, these factors may increase the likelihood that PMOS symptoms emerge at puberty in individuals who would otherwise have remained undiagnosed (45).

The relevance of EGWG to PMOS lies in the convergence of metabolic stress, placental dysfunction, inflammatory imbalance, and androgen availability on the tissues that later define the syndrome: the ovary, hypothalamus, adipose tissue, pancreas, liver, and skeletal muscle. We now move from the maternal environment to the specific programming of the fetal ovaries and hypothalamic-pituitary-ovarian (HPO) axis, where susceptibility to PMOS may become embedded before clinical symptoms appear (43).

## Developmental programming of the ovary and HPO axis

The ovary and HPO axis develop in concert with maternal energy balance. They develop through the coordination of six distinct developmental processes: germline cell migration, folliculogenesis, somatic cell differentiation, stromal modification, neuroendocrine maturation, and the onset of gonadotropin responsiveness (13). Accordingly, the early-life risk for developing PMOS should be viewed as arising from disrupted endocrine organization during gestation rather than beginning once menstrual irregularities or other symptoms appear after menarche (45).

### Fetal folliculogenesis and ovarian stromal remodeling

Fetal ovarian development begins with generation of the germ-cell pool and proceeds through germ-cell proliferation, meiosis initiation, nest breakdown, and primordial follicle formation (49). The fetal ovary will respond to excess maternal nutrition and lipid. The amount of nutrient and lipid provided by a mother could impact the level of reactive oxygen species (ROS), the amount of ROS produced relative to the antioxidant defense system, and the number of cells within the stroma (50). Therefore, a developmental perspective on this would suggest that EGWG does not just predispose a child to be obese at some point in their lives but also may affect how the developing fetus’s ovarian tissue responds to different types of metabolic and gonadotropin signals throughout its lifetime (51).

### Granulosa theca cell signaling and steroidogenic reprogramming

The endocrine function of the ovary depends on close coordination between granulosa and theca cells. Luteinizing hormone stimulates theca cells to produce the precursor for androgen. Follicle-stimulating hormone stimulates granulosa cell conversion of androgen into estrogen via aromatization. Disruption in communication between theca cells that over-produce androgen precursors (and possibly the failure of the granulosa cells to mature), leads to a disruption in normal follicular development and maturation. As a result, a PMOS follicle fails to develop towards selection/ovulation (48).

Pre-reproductive cell interactions could be altered by prenatal metabolic disturbances. Excessive gestational weight gain has been shown to expose the fetus to high levels of insulin, lipids, pro-inflammatory mediators, and possibly greater free androgen, owing to insulin-mediated reductions in sex hormone-binding globulin (SHBG). These maternal exposures may cause an alteration in fetal ovarian steroidogenic enzymes, androgen receptors’ ability to bind androgens, granulosa cell metabolism and possibly a shift from follicular growth to atresia (43). There is experimental evidence supporting that androgen signaling plays an important role in developing ovaries, since androgen receptor activity in granulosa cells is crucial to proper follicular maturation and ovulation. Since there is an association with reduced or increased androgen signaling affecting follicular processes, it follows that the timing, quantity and site of androgen exposure will determine whether effects on follicular development occur (52).

### AMH dysregulation, follicular arrest, and ovarian reserve alteration

Anti-Müllerian hormone (AMH) is a key regulator of follicular recruitment and ovarian reserve dynamics. In Polycystic Ovary Syndrome (PMOS), AMH levels are often elevated due to an increase in the number of small-growing follicles as well as changes in the activity of granulosa cells. Elevated AMH serves as a biomarker for PMOS but it also plays an active role in PMOS disease biology by preventing further follicular recruitment, promoting follicular arrest, and modulating hypothalamus/pituitary signaling (53). Mechanistic reviews published recently describe AMH as a critical molecular pathway linking PMOS/PCOS pathogenesis. Furthermore, recent studies indicate that high maternal plasma AMH concentrations during pregnancy in mothers diagnosed with PCOS could result in alterations in fetal development via interaction with placental aromatase and androgens (54).

The combination of EGWG and maternal PMOS will expose the fetus to a combination of changes in AMH signaling; changes in the availability of androgens (and therefore their bioactivity); changes in insulin sensitivity; and changes in placental metabolism (53). Experimental studies in mice suggest that elevated maternal AMH during pregnancy can alter the maternal and fetal endocrine environment and lead to PCOS-like reproductive and neuroendocrine traits in female offspring. These findings provide a plausible pathway through which maternal endocrine status could influence offspring reproductive development (54). However, whether this mechanism contributes to PMOS risk in humans need further investigation.

### Hypothalamic GnRH pulse programming and LH hypersecretion

The hypothalamic-pituitary-ovarian (HPO) axis regulates reproductive endocrine function: pulsatile gonadotropin-releasing hormone (GnRH) secretion from the hypothalamus drives pituitary LH and FSH release, which in turn controls ovarian steroidogenesis and follicular development. Many phenotypic features of PCOS reflect an increased GnRH pulse frequency, enhanced LH secretion relative to FSH, and an ovary driven toward excess androgen production, all of which can be exacerbated by insulin resistance and reduced progesterone negative feedback (55).

Prenatal androgen-exposed mice are among the best experimental models for demonstrating altered reproductive neuroendocrine development. Investigators have found that prenatal androgenization results in higher levels of testosterone and LH, irregular estrous cycling, and other neuroendocrine disorders consistent with an increase in GnRH neurons. Likewise, reviews on reproductive neuroendocrine dysfunction in women with polycystic ovary syndrome (PCOS) indicate prenatal androgen models to be among the best systems for determining how early androgen exposure affects permanent changes in the hypothalamus/pituitary regulation. These data indicate that androgen-sensitive programming of the brain may occur before ovarian dysfunction and then become self-reinforcing (26).

EGWG could affect neuroendocrine programming via several mechanisms. First, maternal insulin resistance can alter fetal androgen exposure by decreasing levels of Sex Hormone Binding Globulin (SHBG) thereby increasing the amount of free androgens available. Secondly, inflammatory cytokines and adipokines may disrupt development of hypothalamus, hunger regulation as well as reproductive neuroendocrine pathways. Thirdly, alterations in placental function can modulate fetal nutrient and hormone exposures. Fourth, pre-programmed fat accumulation during early years of life may exacerbate pubertal insulin resistance that can then also stimulate ovary androgen synthesis and therefore create an abnormal gonadotropin feedback loop. Together these factors form a logical biological sequence for how fetal exposures; childhood metabolic development; and pubertal endocrine stimulation may lead to PMOS (56).

**Figure 2** integrates the ovarian, neuroendocrine, metabolic, and placental pathways through which EGWG may shape offspring susceptibility to PCOS (57). Systemic insulin resistance, often arising from obesity-associated adipose tissue dysfunction, leads to compensatory hyperinsulinemia. At the ovarian level, insulin acts synergistically with luteinizing hormone (LH) to stimulate androgen synthesis. Dysregulation of insulin, growth factor, gonadotrophin, and sex steroid signaling may further impair follicular development and ovulation, thereby contributing to key reproductive features of PCOS. Beyond these metabolic and reproductive disturbances, genetic susceptibility and developmental influences may also play important roles in shaping PCOS risk (57).

**Figure 2:**
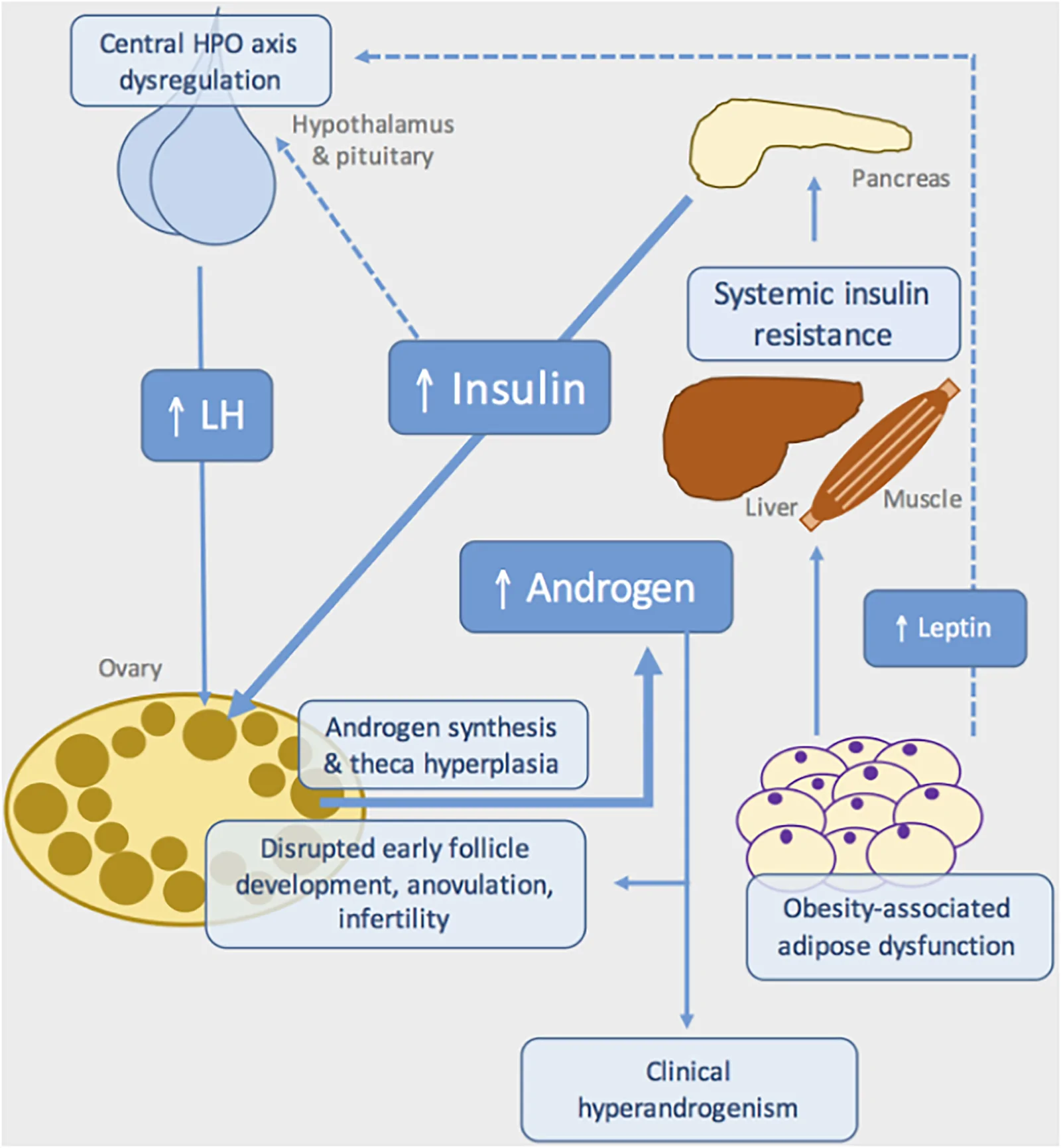
Proposed pathogenic mechanisms in obesity-associated ovarian dysfunction and subfertility.

## Metabolic inheritance and the insulin-androgen feedback loop

EGWG may also contribute to offspring PMOS susceptibility through metabolic inheritance as well as through ovarian and neuroendocrine programming. Metabolic inheritance therefore represents a central pathway through which EGWG shapes offspring PMOS susceptibility (27). During an obese pregnancy the fetus could be exposed to high levels of glucose, lipid, insulin, leptin, pro-inflammatory cytokine and oxidative stress. These signals can affect the development of fetal pancreas, liver, skeletal muscle, adipose tissue and ovary thereby setting up the developmental environment which will lead to increased reproductive endocrine dysfunction from early life on (16).

### Offspring adiposity and programmed insulin resistance

Fetal overnutrition by an obese mother is likely to lead to increased fetal exposure to a variety of nutrients and endocrine signals that regulate the formation of fat depots (adipose tissue), appetite regulation (appetite regulating circuitry) in the brain, insulin sensitive tissues (skeletal muscle and liver) and energy balance regulatory systems (33). Such programming is unlikely to have direct effects on PMOS, but it could lower the threshold at which children or adolescents develop insulin resistance (12).

Offspring obesity should therefore be viewed in two roles: as a developmental endpoint reflecting excess fetal fat deposition, and as a mediator of further disease, since increased body fat worsens insulin resistance and lowers SHBG, raising free testosterone and reinforcing the metabolic-endocrine cycle that underlies the progression of PCOS (58). For those adolescent females who already have some level of metabolic impairment; the entry into puberty may represent the point at which a previously ‘programmed’ risk becomes clinically apparent through symptoms such as hyper-androgenism, amenorrhea, oligomenorrhea, hirsutism and/or acne (43).

### Pancreatic β-cell stress, hepatic metabolism, and adipose dysfunction

Genetically or environmentally induced insulin resistance depends on coordinated functional changes across several major metabolic organ systems. Nutrient abundance during pregnancy could potentially result in an increase in β-cell mass and/or β-cell responsiveness leading to increased levels of insulin postnatally, thereby maintaining blood glucose homeostasis (31). However, although this early adaptation may be protective against acute elevations in blood glucose, chronic elevation in insulin demand will likely contribute to β-cell dysfunction, glucose intolerance, and eventually other forms of metabolic disease (42).

The liver plays an equal role. High-fat intake and EGWG during pregnancy may program the liver to accumulate lipid, produce inflammatory signals, and become less insulin-sensitive. Insulin resistance within the liver can also significantly relate to PMOS since it is where glucose metabolism, lipid storage/handling occur and sex hormone binding globulin (SHBG) is synthesized (33). Since SHBG synthesis is suppressed by the increased levels of insulin that are seen with compensatory hyperinsulinemia, the circulating level of free testosterone will increase and thus increase the bioavailability of androgens regardless of whether or not there is a primary defect of the ovaries (56).

### Insulin-mediated ovarian androgen excess

Insulin resistance may lead to compensatory hyperinsulinemia as the pancreas increases insulin secretion to maintain glucose homeostasis (59). If EGWG contributes to insulin resistance in female offspring, the resulting hyperinsulinemia could provide a mechanistic link between the prenatal metabolic environment and hyperandrogenism later in life. Insulin may enhance androgen production by ovarian theca cells and reduce hepatic production of sex hormone-binding globulin, thereby increasing circulating free androgen levels. However, whether this pathway mediates an association between EGWG and offspring PMOS remains to be established.

Insulin resistance may further amplify androgen excess, forming a bidirectional cycle in which androgen excess promotes insulin resistance and insulin resistance exacerbates androgen excess. EGWG may therefore act on both ends of this loop (43). Obesity may increase susceptibility of offspring to metabolic disturbances (i.e., it programs them for greater vulnerability) as well as increases their endogenous production of androgens due to increased insulin stimulation (27).

### Puberty as the phenotypic unmasking window

Puberty is a critical stage in the development of PMOS. The pubertal transition includes an increase in insulin resistance; maturation of gonadotropin pulsatility; increased production of steroids by the ovaries; and redistribution of fat (8). In a healthy, metabolically resilient adolescent, these transitions stabilize into regular ovulation. By contrast, in an adolescent already programmed with insulin resistance, altered ovarian androgen sensitivity, or changes in hypothalamic-pituitary regulation, the same physiological transition can unmask an existing metabolic liability (48, 62).

EGWG may create susceptibility; however, the child’s adiposity, diet, sleep quality, physical activity, exposure to endocrine-disrupting chemicals, stress responses, and pubertal timing determine whether that susceptibility becomes clinically evident disease (63). Therefore, the insulin-androgen cycle acts as a biological converter through which early metabolic programming produces reproductive endocrine disease. Metabolic inheritance thus offers a coherent model of how EGWG can be a risk factor for PMOS without being its sole determinant (8) (**Table 2**).

**Table 2:** Integrated metabolic programming pathways linking EGWG to offspring PMOS susceptibility.

Programmed pathway	Primary tissue or system affected	Developmental effect	PMOS-relevant outcome
Fetal nutrient oversupply (31)	Pancreas, liver, adipose tissue	Increased insulin demand and altered glucose-lipid handling	Hyperinsulinemia and metabolic vulnerability
β-cell programming (42)	Pancreatic islets	Compensatory insulin secretion and later β-cell stress	Increased risk of insulin resistance and dysglycemia
Hepatic programming (43)	Liver	Altered lipid metabolism, reduced insulin responsiveness, lower SHBG tendency	Increased free androgen bioavailability
Adipose programming (60)	White adipose tissue and appetite circuits	Adipocyte expansion, leptin resistance, inflammatory adipokine profile	Obesity-associated insulin resistance and reproductive disruption
Skeletal muscle insulin resistance (19)	Skeletal muscle	Reduced peripheral glucose uptake	Compensatory hyperinsulinemia
Ovarian metabolic sensitivity (48)	Theca and granulosa cells	Increased responsiveness to insulin and LH signaling	Androgen excess and follicular arrest
Pubertal metabolic stress (61)	HPO axis, adipose tissue, ovary	Physiological insulin resistance superimposed on programmed vulnerability	Menstrual irregularity and hyperandrogenism
Postnatal obesogenic reinforcement (26)	Whole-body metabolic network	Amplification of prenatal metabolic programming	Persistent PMOS phenotype and cardiometabolic risk

## Epigenetic and intergenerational endocrine memory

A central implication of the EGWG hypothesis is that it may create a long-lasting endocrine memory in offspring exposed to an obesogenic, androgen-disrupted gestational environment. Genetic predisposition is unlikely to be the sole mechanism by which PMOS susceptibility is inherited (27). Rather than solely inheriting changes in DNA sequence, offspring from mothers who are obese may inherit environmental responsive epigenetic regulation such as DNA methylation, histone modification, chromatin accessibility, microRNAs (non-coding RNAs), mitochondrial function, and/or germline associated signals as a direct result of the mother being exposed to conditions that promote insulin resistance, chronic low-grade inflammation and elevated levels of androgens (9).

### Intergenerational exposure and transgenerational inheritance

Distinguishing intergenerational effects from transgenerational inheritance requires identifying which generations are directly exposed. During an F0 pregnancy, the maternal metabolic and endocrine environment may affect the developing F1 fetus and, potentially, the primordial germ cells within that fetus that will later give rise to F2 (25). An F0 female who becomes obese develops insulin resistance, inflammation and elevated androgens; these conditions expose her F1 offspring (embryo/fetus) as well as the embryonic primordial germ cells (PGCs), which will be part of the oocytes/eggs for future reproduction by the F2 females. Thus, either reproductive or metabolic abnormality seen in the F1 and F2 can be due to direct developmental exposure from the mother rather than being evidence of transgenerational inheritance (64).

The difference between these two concepts is especially important for researchers looking into PMOS as multigenerational similarity is typically discussed without differentiating between maternal-fetal programming and germline transmission (65). Demonstrating that a pregnant mother’s obesity influences her offspring’s fat mass, insulin sensitivity, ovary function, or puberty timing in their first generation is an important clinical finding; however, this alone does not confirm transgenerational inheritance (66).

### DNA methylation and chromatin remodeling in reproductive-metabolic tissues

DNA methylation is one of the best-characterized mechanisms by which early-life exposures produce lasting gene-regulatory effects. Obesity and over nutrition of the mother may cause alterations in the fetal/neonate phenotype by changing the methylation patterns at certain developmental stages (67). The changes within those same windows, in a framework related to PMOS, would be most relevant if occurring in the tissues regulating the pathways for ovarian steroidogenesis, insulin signaling, adipogenesis, hepatic metabolism, hypothalamus function, or gonadotrophin negative feedback (68).

Experimental models of PMOS provide essential evidence that altered methylation can be an important mechanism of endocrine programming. The association of prenatal androgen exposure to PMOS-like reproductive and metabolic phenotypes through multiple generations and their potential as molecular mediators of transgenerational inheritance is well-documented (69). Epigenetic marks such as chromatin remodeling and histones could potentially increase the stability of gene expression patterns (i.e., making genes either accessible for transcription or repressible) due to the temporal/tissue specificity of these epigenetic modifications. Thus, the same maternal androgenic insult could result in entirely disparate effects based on its ability to modify the germline, granulosa cells, theca cells, adipose cells, liver cells, or hypothalamic neurons (70).

### Non-coding RNAs, mitochondrial dysfunction, and germline transmission

MicroRNAs, long non-coding RNAs, and other small RNAs carried in gametes can modulate gene expression without altering DNA sequence (68). These non-coding RNAs may mediate PMOS-related programming of steroid hormone biosynthesis, insulin signaling, adipose tissue inflammation, mitochondrial function, ovarian follicular development, and, ultimately, the hypothalamic-pituitary-ovarian (HPO) axis (71).

The possibility that PMOS is clinically expressed in females yet transmitted through both female and male germlines is especially pertinent here. Recent experimental evidence indicates that males can transmit metabolic and reproductive risk through sperm-derived small non-coding RNAs, with the phenotype appearing mainly in female descendants (72–74).

### Evidence from prenatal androgen and maternal high-fat diet models

Animal models continue to be the most effective experimental system to determine if EGWG and androgen excess will program PMOS-like features. Exposure of a developing rodent, sheep or related model to prenatal androgens can lead to abnormalities in cycle length, higher than normal levels of circulating androgen, abnormal LH secretion, insulin insensitivity, increased fat storage and morphologic alterations in ovaries similar to those seen with PMOS (75). Later-generation studies provide additional evidence that endocrine disruption can persist after direct fetal exposure has passed, because many cases of reproductive metabolic syndrome have been linked to changes in DNA methylation or candidate genes (26).

Maternal high-fat (high-calorie) dietary models extend this framework from androgen excess to an obese, obesogenic state of metabolic exposure. Maternal high caloric intake is shown to affect fetal/neonatal phenotypes through epigenetically induced changes in the expression of genes in the pancreas (β cells), adipose tissue, liver, placenta and other metabolic organs (76). Therefore, EGWG could “program” for metabolic components of PMOS susceptibility. Androgenic disrupted effects would be thought to program for reproductive/neuroendocrinological aspects of PMOS susceptibility (26). Thus, when there are both EGWG and PMOS present, the offspring will likely suffer from combined metabolic and androgenic programming.

Before translating these findings to clinical applications, the generational logic of the study must be clarified (**Figure 3**). F3 generation is used to infer true transgenerational inheritance following gestational exposure, because F1 fetus and F2 germline are directly exposed. Candidate molecular carriers should be identified including DNA methylation, histone marks, non-coding RNAs, mitochondrial dysfunction and gamete associated regulatory signals (72).

**Figure 3:**
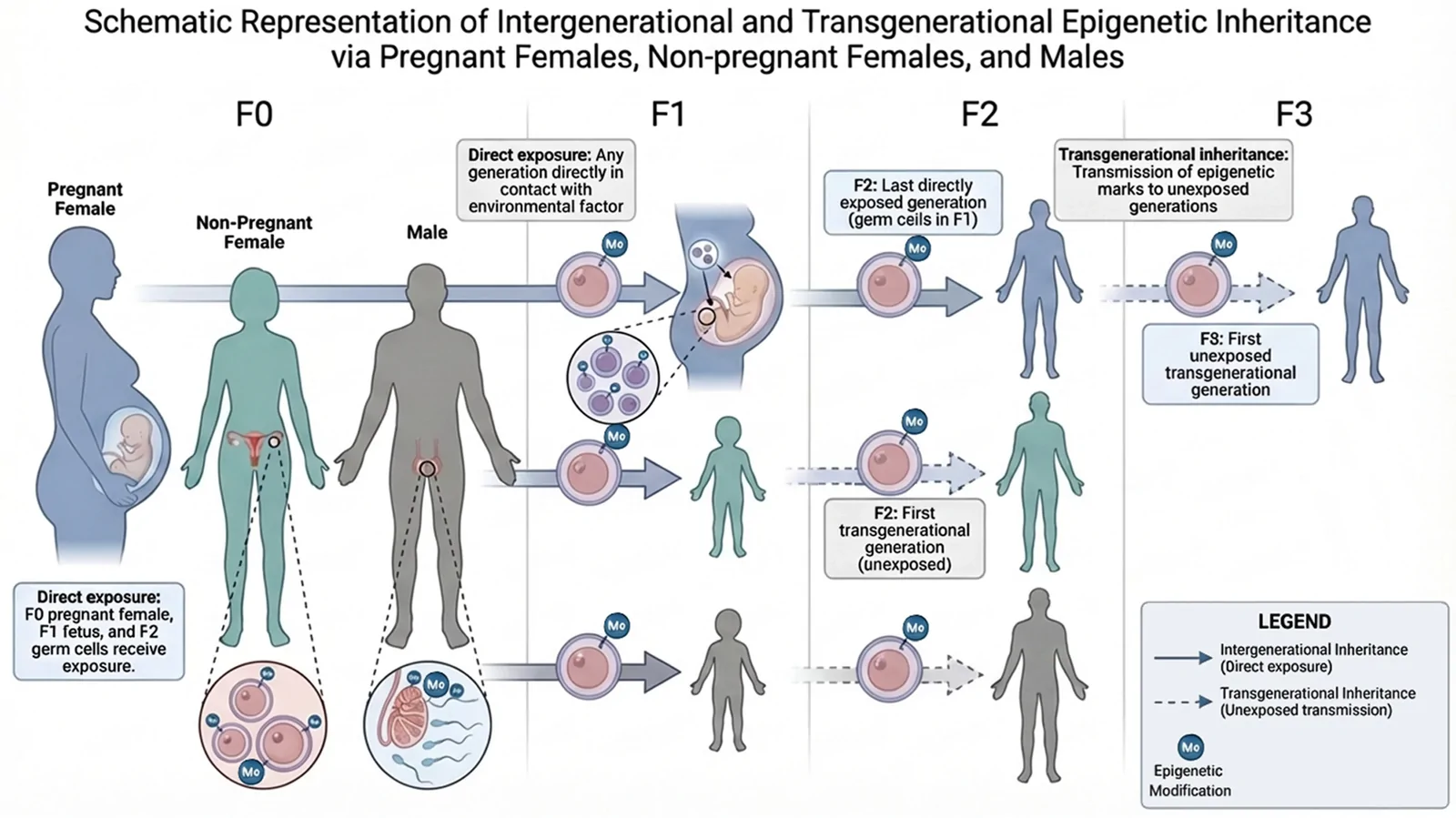
Intergenerational (IEI) and transgenerational epigenetic inheritance (TEI) across maternal and paternal exposure scenarios. Exposure of a pregnant female directly affects the mother (F0), the developing fetus (F1), and the germ cells within that fetus that will form F2, constituting IEI. Consequently, the first unexposed generation, and thus the first in which TEI can be assessed, is F3. In contrast, exposure of a non-pregnant female affects only the germ cells, producing IEI in F1; TEI can therefore first be evaluated in F2. Similarly, direct exposure of males affects their germ cells (F1), making F2 the first unexposed generation in which TEI may be observed.

Overall, the data on epigenetics and the germline provide further biological basis for the EGWG hypothesis; however, they do not establish a proven case of transgenerational inheritance in humans (9). The most defensible interpretation is that this evidence indicates an increased developmental risk of metabolic, ovarian, neuroendocrine, and germline dysfunction in offspring exposed to EGWG or androgen excess, which may manifest as PMOS after puberty if combined with later metabolic stress (27) (**Table 3**).

**Table 3:** Evidence hierarchy for key EGWG-to-PMOS programming claims.

Programmed pathway/claim	Primary evidence type	Mechanism	Outcome	Strength of inference
Prenatal androgen programming	Animal models	Altered GnRH/LH and ovarian steroidogenic signalling	PMOS-like reproductive phenotype	Directly demonstrated (animal); plausible (human)
Maternal high-fat diet and overnutrition	Animal models and human cohorts	Pancreatic, hepatic, adipose, and ovarian programming	Metabolic vulnerability and hyperandrogenism	Direct (animal); observational (human)
Epigenetic and germline transmission	Animal germline models	DNA methylation, histone marks, small non-coding RNAs	Multigenerational phenotype	Direct (animal); not established in humans
Placental dysfunction	Human and animal studies	Inflammation, oxidative stress, altered steroid metabolism	Modified fetal exposure	Direct (human and animal)
Insulin-androgen feedback loop	Human and animal studies	Hyperinsulinemia lowers SHBG and stimulates theca cells	Hyperandrogenism	Direct (human); mechanistic convergence

## Clinical and translational significance

The mechanisms described here have practical implications for the management of PMOS. These mechanisms demonstrate that PMOS is a disorder whose risk can be influenced throughout one’s reproductive life cycle; prior to conception, while pregnant and while developing into a young adult (77). Therefore, if EGWG influences the developmental vulnerability of offspring via alterations to placental function, metabolic systems, ovarian function, neuroendocrine function and epigenetics, then preventing such developmental vulnerabilities cannot solely involve treating the menstrual irregularities, fertility issues, and hyperandrogenic symptoms associated with PMOS once they are diagnosed (78). This approach also aligns with contemporary recommendations regarding PMOS that emphasize comprehensive assessments and provide evidence-based support for reducing cardiovascular and metabolic risks, creating personalized lifestyles, and providing continuous lifetime care versus a focus on solely ovarian disease management (79).

### Gestational metabolic optimization and PMOS risk counseling

The preconception period and early pregnancy offer opportunities to assess and address maternal metabolic risk during critical stages of embryonic and placental development (80). For women with PMOS, preconception assessment should consider weight and adiposity, blood pressure, glycemic status, lipid profile, reproductive history, and family history of diabetes. A 75-g oral glucose tolerance test should be considered when planning pregnancy; if it has not been performed before conception, testing can be offered at the first prenatal visit and repeated at 24–28 weeks. Lifestyle factors, including nutrition, physical activity, smoking, and sleep, should also be addressed. This assessment can guide individualized care for established maternal and pregnancy risks, without implying that such care has been shown to prevent PMOS in offspring (81).

Counseling should frame preconception weight management around metabolic and reproductive health. During pregnancy, the focus should shift to appropriate gestational weight gain and evidence-based care. Discussions may address nutrition, physical activity, sleep, mental well-being, and the management of glycemic and lipid risk factors. In some individuals, obesity-associated insulin resistance and compensatory hyperinsulinemia can reduce SHBG level, increasing circulating free androgens. Whether improving these maternal factors alters the fetal endocrine environment or reduces offspring PMOS risk, however, remains further validation (82).

### Gestational monitoring of metabolic, inflammatory, and placental risk

Screening for hyperglycemia before conception and again in early pregnancy, with retesting at the recommended mid-pregnancy time point, may be especially important to prevent PMOS (83). Antenatal care should include monitoring of blood pressure, gestational weight gain, and glycemic status, with assessment of fetal growth and screening for sleep-disordered breathing when clinically indicated. Lipid abnormalities or inflammatory comorbidities should be evaluated according to the patient’s history and clinical findings rather than treated as universal features of EGWG. Beyond supporting fetal growth, the placenta regulates nutrient transfer and has endocrine and metabolic functions that may modify fetal exposure to the maternal environment. How EGWG relates to these placental processes remains incompletely understood. Placental inflammation, mitochondrial function, and steroid metabolism are important research targets, but they are not routinely measured in clinical care. Monitoring decisions should therefore follow established maternal and fetal risk assessments, rather than presumed placental abnormalities (84).

### Early life surveillance of offspring adiposity, puberty, and androgenic signs

Although EGWG alone does not predict PMOS or warrant intensified long-term surveillance, routine pediatric care offers opportunities to monitor daughters’ growth, weight trajectory, and pubertal development. If concerns arise, clinicians may assess menstrual regularity after menarche, persistent or severe acne, hirsutism, androgenic hair loss, and signs of metabolic dysfunction such as acanthosis nigricans. These findings should be interpreted in light of the individual’s age and stage of pubertal development (85).

Diagnosing PMOS in adolescence requires caution because menstrual irregularity and acne may be part of normal pubertal development. Throughout childhood and adolescence, families and clinicians can support general health through balanced nutrition, regular physical activity, adequate sleep, and emotional support, while avoiding weight-stigmatizing messages. Puberty is an important period for assessment: the normal, temporary decline in insulin sensitivity may make underlying metabolic vulnerability more apparent. Nevertheless, these measures should be presented as support for overall health, not as proven strategies to prevent PMOS in daughters born after pregnancies with EGWG (62).

### Ethical framing: prevention without maternal blame

Communicating the transgenerational programming model of obesity requires an ethical framing. Both EGWG and PMOS arise from many interacting genetic, biological, environmental, and endocrine factors. EGWG should therefore be viewed as a modifiable factor that influences the likelihood of developing PMOS rather than as its cause (86).

The relevance for translational research is found by relating biological mechanisms to clinical timing. While the proposed model does not suggest that elimination of PMOS is possible solely through maternal weight management, it suggests that the intergenerational reproductive-metabolic risks may be reduced when there is earlier, integrated and less stigmatized care.

## Limitations and future directions

This review has several limitations. Human studies vary in their definitions of EGWG, diagnostic criteria for PMOS/PCOS, and assessment of offspring outcomes. Most are observational and cannot fully separate potential intrauterine effects from shared genetic susceptibility, maternal PMOS, gestational diabetes, socioeconomic factors, or postnatal influences such as diet, adiposity, and pubertal development. Animal models offer opportunities to test mechanisms involving specific exposures, including prenatal androgen excess and maternal high-fat diets, but they reproduce only selected features of human PMOS. Moreover, after an exposure during an F0 pregnancy, both the F1 fetus and the germ cells that may give rise to F2 can be directly affected. Phenotypes observed in F1 or F2 therefore cannot, on their own, establish transgenerational inheritance. Together, these limitations prevent firm causal conclusions about EGWG and offspring PMOS risk.

Future research should prioritize prospective mother–daughter cohorts beginning before conception or early in pregnancy and continuing into adulthood. Repeated maternal assessments should capture PMOS status, prepregnancy body composition, gestational weight gain, glycemic and lipid measures, androgen levels, inflammatory markers, medication use, and pregnancy complications. Placental transcriptomic, epigenomic, proteomic, metabolomic, lipidomic, and single-cell analyses could help identify pathways linking the maternal environment with offspring development. Such findings should be treated as mechanistic evidence; any proposed placental biomarkers would require independent validation before being used to predict later disease. Offspring follow-up should extend beyond birth outcomes to repeated assessments of growth and body composition, metabolic health, pubertal development, menstrual function, and clinical or biochemical hyperandrogenism. Together, these longitudinal data could help distinguish the contribution of gestational exposures from maternal PMOS, inherited susceptibility, and postnatal influences.

Animal studies can examine how maternal diet, androgen levels, glucose regulation, and the offspring’s postnatal environment contribute to PCOS-like traits. To investigate transgenerational inheritance after exposure during pregnancy, studies must assess the first unexposed generation (F3) and test whether effects are transmitted through the germline. Intervention studies should evaluate whether care before and during pregnancy, postpartum support, or early-life support improves offspring health. These approaches should be discussed without blaming mothers or claiming that they prevent PMOS before this has been demonstrated.

## Conclusion

PMOS can be understood as a disorder involving reproductive and metabolic systems, with susceptibility potentially shaped during development. This review considers whether EGWG is associated with maternal metabolic conditions that may influence offspring development. Proposed pathways involve placental nutrient and hormone regulation, fetal ovarian and neuroendocrine development, and later metabolic health. However, EGWG does not necessarily indicate that these conditions are present, and evidence that it independently increases offspring PMOS risk remains limited.

The proposed model has no single cause. Inherited susceptibility, maternal health, prenatal development, and postnatal and pubertal factors may all contribute to PMOS risk. Experimental findings suggest possible roles for insulin and androgen signaling and lasting changes in gene regulation, but whether EGWG produces these effects in humans has not been established.

This developmental perspective supports attention to metabolic health before and during pregnancy and to offspring growth and pubertal health through routine care. These measures may improve health, but they should not be presented as proven ways to prevent PMOS. Counseling should avoid blaming mothers: gestational weight gain is shaped by biological, social, and healthcare factors, and it does not determine an individual child’s future health.
